# Three-dimensional structures of two heavily N-glycosylated *Aspergillus* sp. family GH3 β-d-glucosidases

**DOI:** 10.1107/S2059798315024237

**Published:** 2016-01-28

**Authors:** Jon Agirre, Antonio Ariza, Wendy A. Offen, Johan P. Turkenburg, Shirley M. Roberts, Stuart McNicholas, Paul V. Harris, Brett McBrayer, Jan Dohnalek, Kevin D. Cowtan, Gideon J. Davies, Keith S. Wilson

**Affiliations:** a York Structural Biology Laboratory, Department of Chemistry, The University of York, York YO10 5DD, England; b Novozymes Inc., 1445 Drew Avenue, Davis, CA 95618, USA

**Keywords:** cellulose degradation, biofuels, glucosidase, N-glycan, glycoblocks

## Abstract

The industrial conversion of cellulosic plant biomass into useful products such as biofuels is a major societal goal. These technologies harness diverse plant degrading enzymes, classical exo- and endo-acting cellulases and, increasingly, cellulose-active lytic polysaccharide monooxygenases, to deconstruct the recalcitrant β-d-linked polysaccharide. A major drawback with this process is that the exo-acting cellobiohydrolases suffer from severe inhibition from their cellobiose product. β-d-Glucosidases are therefore important for liberating glucose from cellobiose and thereby relieving limiting product inhibition. Here, the three-dimensional structures of two industrially important family GH3 β-d-glucosidases from *Aspergillus fumigatus* and *A. oryzae*, solved by molecular replacement and refined at 1.95 Å resolution, are reported. Both enzymes, which share 78% sequence identity, display a three-domain structure with the catalytic domain at the interface, as originally shown for barley β-d-glucan exohydrolase, the first three-dimensional structure solved from glycoside hydrolase family GH3. Both enzymes show extensive N-glycosylation, with only a few external sites being truncated to a single GlcNAc molecule. Those glycans N-linked to the core of the structure are identified purely as high-mannose trees, and establish multiple hydrogen bonds between their sugar components and adjacent protein side chains. The extensive glycans pose special problems for crystallographic refinement, and new techniques and protocols were developed especially for this work. These protocols ensured that all of the d-pyranosides in the glycosylation trees were modelled in the preferred minimum-energy ^4^ *C*_1_ chair conformation and should be of general application to refinements of other crystal structures containing O- or N-glycosylation. The *Aspergillus* GH3 structures, in light of other recent three-dimensional structures, provide insight into fungal β-d-glucosidases and provide a platform on which to inform and inspire new generations of variant enzymes for industrial application.

## Abstract 2

The three-dimensional structures of two industrially important family GH3 β-d-glucosidases from *A. fumigatus* and *A. oryzae* are reported at 1.95 Å resolution. Both enzymes show extensive N-glycosylation. The extensive glycans pose special problems for crystallographic refinement, and new techniques and protocols were developed especially for this work.

## 1. Introduction   

**Figure 1:**
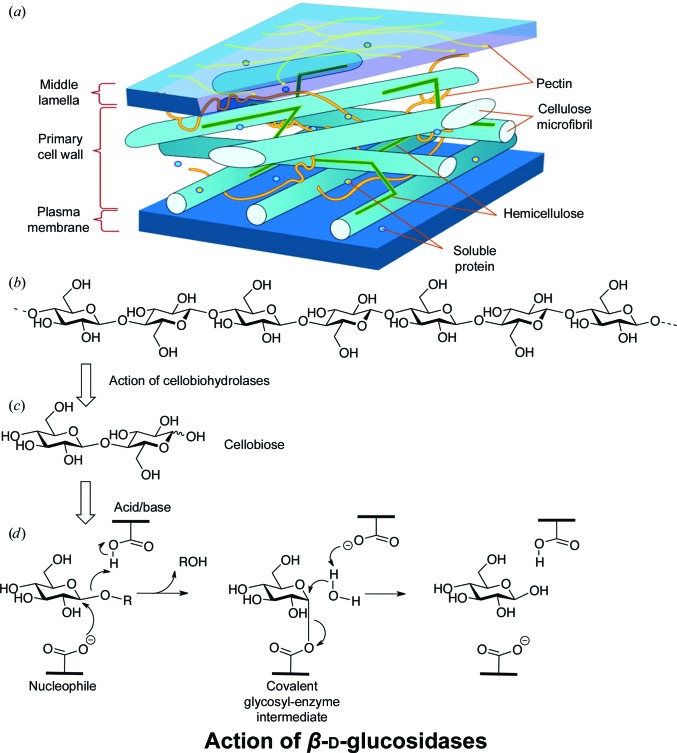
Cellulose: structure and breakdown. (*a*) Generic representation of the plant cell wall (taken from https://commons.wikimedia.org/wiki/File:Plant_cell_wall_diagram.svg). (*b*) Structure of a single β-1,4-d-glucan chain. (*c*) Structure of the disaccharide cellobiose generated by the action of cellobiohydrolases. (*d*) Mechanism of a family GH3 retaining β-d-glucosidase; hydrolysis occurs *via* a covalent glycosyl-enzyme intermediate.

β-d-Glucosidases (EC 3.2.1.21) are classical glycoside hydrolases (reviewed in Davies *et al.*, 1995 ▸; Henrissat & Davies, 1997 ▸) that catalyse the hydrolysis of the nonreducing terminal glucose from β-linked d-gluco-oligosaccharides and aryl-β-d-glucosides. In the CAZy (Henrissat & Davies, 1997 ▸; Lombard *et al.*, 2014 ▸) sequence-based classification of carbohydrate-active enzymes, β-d-glucosidases are found in families GH1, GH3, GH5, GH9, GH30 and GH116. With the exception of family GH9, all of the β-d-glucosidases are retaining enzymes, in which a covalent glycosyl-enzyme intermediate is formed and subsequently hydrolysed *via* an oxocarbenium-ion-like transition state. Such a mechanism demands two crucial catalytic residues, a nucleophile and an acid/base (Fig. 1 ▸), both of which are typically enzyme-derived carboxylates (such mechanisms are reviewed in Vocadlo & Davies, 2008 ▸; Davies *et al.*, 2012 ▸), as discussed further below.

The primary biotechnological importance of β-d-glucosid­ases is their key role in cellulose degradation (Fig. 1 ▸). The enzymatic degradation of cellulose is playing an increasing role in society through applications in the paper and textile industries, in detergents and, most notably in recent times, in biofuel production (reviewed in, for example, Ragauskas *et al.*, 2006 ▸; Himmel *et al.*, 2007 ▸; Gilbert *et al.*, 2008 ▸). Cellulose degradation involves the initial attack of both lytic polysaccharide monooxygenases (Quinlan *et al.*, 2011 ▸; Harris *et al.*, 2014 ▸) and classical endo-acting endoglucanases that produce free chain ends upon which the processive cellobiohydrolases CBH I and CBH II act (recently reviewed in Horn *et al.*, 2012 ▸). CBH I and CBH II release the disaccharide cellobiose (the β-1,4-linked disaccharide of glucose) as product and both enzymes suffer from considerable product inhibition. β-d-Glucosidases therefore have a central role both in the production of glucose and, crucially, in the alleviation of the product inhibition of CBH I and CBH II during the cellulose-degradation process itself (see, for example, Xin *et al.*, 1993 ▸; Berlin *et al.*, 2007 ▸).

Family GH3 of the CAZy classification includes enzymes with a diverse array of specificities for which many exemplar three-dimensional structures are known (at the time of submission, 20 different GH3 structures were available; http://www.cazy.org/GH3_structure.html). Structures include β-d-glucan exohydrolase, the first three-dimensional structure solved for this family (Varghese *et al.*, 1999 ▸), as well as diverse β-d-glucosidases, β-d-xylosidases (Rojas *et al.*, 2005 ▸) and β-*N*-acetyl-hexosaminidases. With the exception of the unusual *N*-acetyl-hexosaminidases, all of the three-dimensional structures from the GH3 family are multidomain proteins with the active centre at a domain interface and with – highly unusually for glycoside hydrolases – key catalytic residues contributed from different domains.

For the classical β-d-glucanase members of GH3 (the *N*-acetyl-hexosaminidases are a clear exception; Stubbs *et al.*, 2008 ▸; Bacik *et al.*, 2012 ▸) the N-terminal (β/α)_8_ barrel houses the nucleophile whilst the α/β-sandwich B domain houses the catalytic acid. Complicating matters further, the sequence diversity in the family, coupled to the observation that the acid/base and nucleophile are derived from different domains, has made it difficult to predict the acid/base residue from sequence alone. In this context, mutagenesis and kinetic studies with substrates of different leaving-group ability (Thongpoo *et al.*, 2013 ▸) have been crucial in assigning the acid/base residue of the *Aspergillus* GH3 enzymes that are the object of the present study.

Here, we present the three-dimensional structures of two industrially relevant family GH3 β-d-glucosidases from *A. oryzae* (*Ao*βG) and *A. fumigatus* (*Af*βG). The structures, which are similar to that reported for the *A. aculeatus* GH3 enzyme (*Aa*βG; PDB entries 4iib, 4iic, 4iid, 4iie, 4iif, 4iig and 4iih; Suzuki *et al.*, 2013 ▸), possess the canonical GH3 three-domain structure with an active centre consistent with the recent assignment by the Brumer group (Thongpoo *et al.*, 2013 ▸). All three proteins form dimers in the crystal. In common with *Aa*βG, both enzymes display significant, extended N-glycosyl­ation that spawns from structurally conserved sites, with varying degrees of definition. Most of the high-mannose tree structures show a remarkable degree of overlap that can be verified at all levels: either in one particular structure (by non­crystallographic symmetry superposition) or even across the *Af*βG, *Ao*βG and *Aa*βG structures (superposition by *SSM*; Krissinel & Henrick, 2004 ▸). Also, the conformations of all the d-pyranoside residues in the glycosylation trees in the present structures have been restrained to lie in the preferred ^4^ *C*_1_ chair conformation. As has recently been demonstrated (Agirre, Davies *et al.*, 2015 ▸), the refinement of pyranose sugar structures poses special problems at lower resolutions owing to a lack of appropriate restraints. Just as the indole group in a tryptophan must be restrained to be kept planar at resolutions which are pooer than atomic, the pyranose conformation easily deviates from the minimum-energy conformation at lower resolutions, resulting in conformational anomalies that currently affect nearly 20% of all N-glycan d-pyranosides in the PDB and 25% of their PDB_REDO (Joosten *et al.*, 2014 ▸) equivalents.

## 2. Experimental procedures   

### 2.1. Gene cloning, expression and protein purification   

The *A. oryzae* gene encoding the GH3 β-d-glucosidase was originally cloned as a cDNA (Schülein & Lehmbeck, 2002 ▸) and was subsequently subcloned and expressed as a heterologous gene in *A. oryzae* as described previously (Lamsa *et al.*, 2004 ▸). Larger scale (2 l) fermentation was performed as described in Example 11 of McBrayer *et al.* (2011 ▸). Approximately 835 ml of fermentation broth was concentrated to 150 ml using a Pall Filtron tangential flow ultrafiltration device fitted with a 10 kDa cutoff polyether­sulfone membrane. This was further concentrated to 50 ml using an Amicon ultrafiltration device fitted with a 76 mm PM10 10 kDa cutoff membrane. The concentrated material was loaded onto a 500 ml Q Sepharose Big Beads (GE Healthcare) column equilibrated with 20 m*M* Tris–HCl pH 8.0 (buffer *A*). The column was washed with 1.5 column volumes of buffer *A* and proteins were eluted with a linear gradient from 0 to 1.0 *M* NaCl in buffer *A* over five column volumes at a flow rate of 20 ml min^−1^. UV-absorbing material eluted as a moderately broad peak centred at about 290 m*M* NaCl. Fractions with a band of the correct size as judged by SDS–PAGE were pooled and buffer-exchanged by ultrafiltration into 50 m*M* Tris–HCl pH 8.0. Judging by SDS–PAGE, the purity of *Ao*βG was greater than 90%.

The *A. fumigatus* gene encoding the GH3 β-d-glucosidase was cloned as a genomic sequence, subcloned into an expression vector, transformed into *A. oryzae* Jal250 and expressed on a 2 l scale as described previously (Teter *et al.*, 2005 ▸). The filtered broth was desalted and buffer-exchanged with 20 m*M* Tris–HCl pH 8.5 using a Pall Filtron tangential flow concentrator equipped with a 10 kDa cutoff polyether­sulfone membrane. The protein was loaded onto a 75 ml Q Sepharose High Performance column (GE Healthcare) equili­brated in 20 m*M* Tris–HCl pH 8.5 and bound proteins were eluted with a linear gradient from 0 to 600 m*M* sodium chloride. The *Af*βG fractions were pooled based on SDS–PAGE analysis, and pooled fractions were concentrated by ultrafiltration using a Vivaspin 20 (Sartorius Stedim Biotech) with a 10 kDa cutoff membrane. The concentrated protein was loaded onto a Superdex 200 HR 26/60 column (GE Healthcare) equilibrated with 20 m*M* Tris–HCl, 150 m*M* sodium chloride pH 8.5. The eluted β-d-glucosidase was adjusted to 1.5 *M* ammonium sulfate and applied onto a Phenyl Superose column (HR 16/10, GE Healthcare) equilibrated with 20 m*M* Tris–HCl pH 8.5, 1.5 *M* ammonium sulfate. Bound proteins were eluted with a linear gradient from 1.5 to 0 *M* ammonium sulfate in 20 m*M* Tris–HCl pH 8.5. The pooled fractions were concentrated and desalted into 25 m*M* Tris–HCl pH 8.5 by ultrafiltration using a Vivaspin 20 (Sartorius Stedim Biotech) with a 10 kDa cutoff membrane. As judged by SDS–PAGE, the *Af*βG was approximately 95% pure.

### 2.2. Crystallization, data collection and structure solution   

Both proteins were crystallized using hanging-drop vapour diffusion. *Af*βG was crystallized at 13 mg ml^−1^, mixed in a 1:1 volume ratio with well solution consisting of 21% polyethylene glycol (PEG) 1500, 25% ethylene glycol and 0.1 *M* MIB, a PACT screen buffer (Molecular Dimensions; consisting of sodium malonate, imidazole and boric acid in a 2:3:3 molar ratio) at pH 5.0. A crystal was harvested into liquid nitrogen, without the need for additional cryoprotectant, using a nylon CryoLoop (Hampton Research). Data were collected to 1.95 Å spacing on beamline I24 at Diamond Light Source and were processed using *MOSFLM* (Leslie & Powell, 2007 ▸) and scaled with *AIMLESS* (Evans & Murshudov, 2013 ▸). The space group was *P*2_1_2_1_2_1_. The structure was solved using programs from the *CCP*4 suite (Winn *et al.*, 2011 ▸). Molecular replacement was employed using *MOLREP* (Vagin & Teplyakov, 2010 ▸), with the structure of the *Thermotoga neapolitana* homologue (PDB entry 2x40; Pozzo *et al.*, 2010 ▸) as a search model; structure solution was performed prior to the publication of the structure of the more closely related homologue. The structure was rebuilt using *Coot* (Emsley *et al.*, 2010 ▸) interspersed with maximum-likelihood refinement using *REFMAC* (Murshudov *et al.*, 2011 ▸). The refined model contained a dimer of the protein, which was the most favourable assembly as calculated by *PISA* (Krissinel & Henrick, 2007 ▸), in the asymmetric unit (residues 21–863 of chains *A* and *B*), with each subunit having nine glycosylation sites ranging from 1–11 residues in length (45 and 46 sugar monomers were associated with chains *A* and *B*, respectively), as well as 39 ethylene glycol molecules, seven imidazole molecules and 1527 water molecules. The final *R* and *R*_free_ were 0.15 and 0.17, respectively.

Crystals of *Ao*βG were obtained from drops of protein at 20 mg ml^−1^ mixed in a 1:1 volume ratio with well solution consisting of 30% PEG 400, 0.2 *M* magnesium chloride, 0.1 *M* HEPES pH 7.5. A crystal was harvested as above, and data were collected on the ID14.2 beamline at the ESRF to 1.95 Å resolution and were integrated with *MOSFLM* and scaled with *AIMLESS*. The space group was either *P*2_1_2_1_2_1_ or *P*2_1_2_1_2 (one of the crystallographic axes lay along the rotation axis and unambiguous space-group assignment was not possible from inspection of systematic absences). The structure was solved employing the refined structure of *Af*βG as the search model in space group *P*2_1_2_1_2_1_ and was refined following similar methods to those used for *Af*βG to an *R* and *R*_free_ of 0.22 and 0.25, respectively. The final model contains two protein dimers (*AB* and *CD*, again as calculated by *PISA*) in the asymmetric unit (chain *A* consisting of residues 23–861, *B* of 23–860, *C* of 23–861 and *D* of 23–860), with the following numbers of glycosylation sites: chain *A*, ten sites (with 40 sugar monomers); chain *B*, nine sites (42 sugar monomers); chain *C*, ten sites (41 sugar monomers); chain *D*, nine sites (34 sugar monomers). There are a PEG molecule and a Cl^−^ ion associated with each chain. There are two Mg^2+^ ions in chains *A* and *C* which are coordinated to a water molecule which forms hydrogen bonds to both Asp722 O and NAG1202 O7; two other Mg^2+^ ions interact with waters which are hydrogen-bonded to both Asp558 (in chains *A* and *D*) and NAG1601 O7 in symmetry-related molecules. In addition, there are a PEG molecule and a phosphate ion associated with chain *A*, and 2117 water molecules.

Coordinates and X-ray data for both structures have been deposited in the PDB. Details of X-ray data-quality and structure-refinement statistics are given in Table 1 ▸.

### 2.3. Building and fitting the sugars   

One of the features of these fungal enzymes is the presence of extensive and interacting N-glycans (see below), which posed particular challenges for correct refinement. All sugars forming N- and O-glycans are expected to be in the lowest energy ^4^ *C*_1_ chair conformation, with the exception of a couple of l-pyranoside rings, where the ^1^ *C*_4_ conformation is often preferred. No sugars of the latter type are present in the β-d-glucosidase structures described here. However, when working with poorer than atomic resolution data, most model-building and refinement software do not use energy-minimization techniques, but rather include a set of geometric restraints that approximate the correct chemistry. These restraints define ideal values and their respective acceptable deviations for bond lengths, angles, planes, chiral volumes and torsions, with the first four being the only ones actively used by default in the existing versions of both *REFMAC*5 and *Coot*. While a chair conformation has neither bond length nor angle strain, there are in addition a number of higher energy conformations that also show minimal or no strain. Any refinement process that exclusively minimizes the deviations from ideal bond lengths and angles can lead to sugar models in such higher energy conformations after attempting to fit them to featureless or incomplete electron-density maps, as has recently been demonstrated (Agirre, Davies *et al.*, 2015 ▸).

In the present study, all NAG (*N*-acetyl-β-d-glucosamine), BMA (β-d-mannopyranose) and MAN (α-d-mannopyranose) sugar monomers were imported from dictionaries created with *ACEDRG* into *Coot* and showed the expected initial ^4^ *C*_1_ conformation. *ACEDRG* was used because it has been reported (Paul Emsley, personal communication) to produce geometric targets for bond lengths and angles that approximate well the values expected by *MOGUL* (Bruno *et al.*, 2004 ▸), which approximate real chemistry better than the classic Engh and Huber values (Engh & Huber, 1991 ▸) used to build the *REFMAC*5 monomer library (Vagin *et al.*, 2004 ▸). Torsion restraints had to be activated in order to keep a ^4^ *C*_1_ conformation, but at present *ACEDRG* produces generic torsion values corresponding to the different combinations of hybridizations along the restrained bond (*e.g.* 60° for *sp* ^3^–*sp* ^3^). For this reason, the generic torsion values were replaced by ones measured from the lowest energy conformer, which *ACEDRG* calculates using *RDKIT* (http://www.rdkit.org). Torsion restraints were activated in *REFMAC*5 using a keywords file containing lines beginning with ‘RESTR TORS INCLUDE RESI’ and ending in ‘NAG’, ‘BMA’ and ‘MAN’.

Manual rebuilding was performed between refinement cycles using *Coot* with the same custom library file input as used for *REFMAC*5. Torsion-angle restraints were enabled in the Refinement and Regularization Parameters window.

The stereochemistry and conformation of the sugars were checked between refinement and rebuilding cycles in order to ensure chemical correctness. The software *Privateer* (Agirre, Iglesias-Fernandez *et al.*, 2015 ▸) was used to this effect, but was extended to generate linear glycan descriptions including those presented here (Tables 2 ▸ and 3 ▸) and to produce script files for generating an interactive list of detected issues that could be used in subsequent sessions of model rebuilding with *Coot*, loading maps (calculated from 2*mF*_o_ − *DF*_c_ and OMIT *mF*_o_ − *DF*_c_ coefficients) and activating torsion restraints automatically. *Privateer* is distributed by *CCP*4 starting from the v.6.5 release.

### 2.4. Ion-pair analysis   

Although many available programs are able to list all intrasubunit and intersubunit salt bridges, to the best of our knowledge none of them allows rapid visual inspection. To address this deficiency, we created the *CCONTACTS* program, which follows the convention introduced by Kumar & Nussinov (1999 ▸) for the detection of salt bridges between ion pairs and makes the list of contacts interactively accessible within *Coot* by using either the Python or Scheme scripts produced. The *CCONTACTS* program will be distributed by *CCP*4 in the forthcoming v.7.0 release.

## 3. Results and discussion   

### 3.1. Three-dimensional folds of *Aspergillus* sp. GH3 enzymes   

The structures of *Af*βG and *Ao*βG are very similar to that of *Aa*βG, with each protomer having three domains. The N-terminal domain (domain A; residues 21–357; *Af*βG numbering) consists of a pair of α-helices preceding a (β/α) domain consisting of three antiparallel β-strands, followed by five parallel β-strands each preceded by an α-helix. The second domain (domain B; residues 386–589; *Af*βG numbering) is an α/β sandwich in which three α-helices are stacked against a six-stranded β-sheet consisting of one antiparallel and five parallel β-strands with a pair of α-helices on the opposite side. At the C-terminus there is a fibronectin type III (FnIII) domain (domain C; residues 655–861; *Af*βG numbering), which is comprised of a β-sandwich of two antiparallel β-sheets of three and four strands which lie close to the interface between domains A and B. The first strand of the three-stranded sheet is separated into two shorter β-strands by a short loop, and there are a short α-helix and a double-stranded β-sheet on a loop between strands 5 and 6 close to domain A.

There are loops connecting domains A and B (358–385; *Af*βG numbering) and domains B and C (590–654). There is also a long loop inserted between the first two β-strands of the FnIII three-stranded sheet, which extends around the outer edge of domain A to the other side of the molecule (674–756 in *Af*βG). There is a short section of disordered peptide in the *Af*βG structure (670–673) at the start of this long loop, in a similar position to an unmodelled loop region in the *Aa*βG structure, which is ordered in *Ao*βG.

The domain organization in the crystal structures of *Af*βG and *Ao*βG is very similar to those observed in X-ray structures of the GH family 3 (GH3) β-d-glucosidases TnBgl3B, *Aa*βG, KmBgl1 (from *Kluyveromyces marxianus*; PDB entry 3abz; Yoshida *et al.*, 2010 ▸) and HjCel3A (from *Hypocrea jecorina*; PDB entry 3zyz; Karkehabadi *et al.*, 2014 ▸). In contrast, a small-angle X-ray scattering (SAXS) dummy-atom model calculated for the *A. niger* β-d-glucosidase from GH3 (*An*Bgl1) reveals a more linear molecular arrangement (Lima *et al.*, 2013 ▸), in which the FnIII domain is located away from domains A and B on an extension provided by the linker peptide. Interestingly, TnBgl3B, KmBgl1 and HjCel3A lack an elongated linker region, and although *Af*βG, *Aa*βG and *Ao*βG possess the linker they do not exhibit an extended FnIII conformation in their X-ray structures (and neither do any GH3 structures determined to date). It is probable that the more compact domain arrangements favour better crystal lattice contact formation. In the *Af*βG structure, for example, FnIII residues 762–764 are hydrogen-bonded to Arg45 and Glu49 of domain A in a symmetry-related molecule. The linker itself is hydrogen-bonded to the C-terminal Arg861 (*via* residues Pro726-Asn727) and to the FnIII domain of a symmetry-related molecule (*via* Trp730).

The *An*Bgl1 FnIII domain has been shown in molecular dynamics simulations to be likely to interact with aromatic groups on lignin-type molecules *via* arginine and tryptophan side chains on its surface which mediate stacking interactions (Lima *et al.*, 2013 ▸). Binding to lignin *via* the FnIII domain would locate the β-d-glucosidase catalytic domain close to cellulose in the cell wall, in proximity to other cellulose-digesting enzymes such as endoglucanases and cellobio­hydrolases, with which it acts in concert by degrading cellobiose.

### 3.2. The enzyme dimers   

The subunit–subunit interface within the dimer of the *Af*βG and *Aa*βG structures spans the α/β-sandwich domain and the extended loop between domains A and B. In *Af*βG there are an ethylene glycol (between Arg469 NH1 in both chains) and two imidazole molecules (between Arg387 O on one chain and Gly475 O on the other) forming hydrogen bonds between the subunits. There is a further ethylene glycol molecule, hydrogen-bonded to Gln496 OE1 and NE2, and also bonded *via* a water molecule to Asn434 ND2 in the other chain. In *Ao*βG the four protomers in the asymmetric unit are arranged as two dimers, with the second (chains *C* and *D*) at right angles to the first (chains *A* and *B*), interacting *via* their FnIII domains (chain *A* with *C* and chain *B* with *D*). There are no sugar-mediated interactions in the FnIII dimer–dimer interfaces, which are comprised solely of protein–protein and protein–solvent hydrogen bonds, whilst there are a couple of interactions between glycans at the subunit–subunit interface between chains *A* and *B* and chains *C* and *D* within each dimer, as detailed below.

### 3.3. Active centre   

The active site of the ligand-free structure of *Aa*βG accommodates an acetate ion in the −1 subsite and a molecule of 2-methyl-2,4-pentanediol (MPD) in subsite +1 (subsite nomenclature is discussed in Davies *et al.*, 1997 ▸); in addition, there is an MPD molecule occupying a position equivalent to subsite +4. *Af*βG has two molecules of ethylene glycol in the substrate-binding site, occupying subsites +1 and −1, in molecules *A* and *B*; the former is hydrogen-bonded to Arg99 NH1 and NH2 and the latter to Asp93 OD1 and OD2 and to Lys190 NZ, as well as to the first ethylene glycol molecule. In molecule *A*, several molecules of ethylene glycol occupy the substrate-binding cleft, one of which overlays on the MPD molecule at the +4 subsite when superposed on the *Aa*βG structure. In *Ao*βG there is a PEG molecule bound in the +1 site, hydrogen-bonded to Asp93 OD2 (in three of the four molecules) and/or to Arg99 NH1 (in two of the four protomers in the asymmetric unit). All of the active-site bound ligands in these structures were derived from their respective crystallization mother liquors.

**Figure 2:**
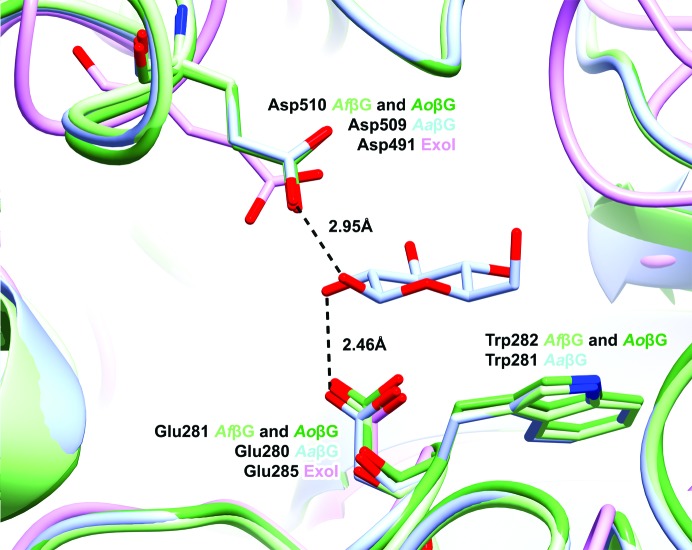
Superposition of the active sites of the enzymes. The catalytic residues proposed for ExoI (PDB entry 1ex1), Asp491 and Glu285 (Thongpoo *et al.*, 2013 ▸), are shown superposed on *Af*βG, *Ao*βG and *Aa*βG (PDB entry 4iig). The structural figures were all produced using *CCP*4*mg* (McNicholas *et al.*, 2011 ▸).

Complexes of *Aa*βG with ligand occupying both the +1 and −1 sugar subsites have been reported (d-glucose in PDB entry 4iig, depicted in Fig. 2 ▸, and thiocellobiose in PDB entry 4iih). In these structures the −1 subsite sugar forms stacking interactions with the side chain of Trp281, and the +1 subsite sugar occupies a cavity bordered by the side chains of Trp68 and Phe305 on one side and stacked against Tyr511 on the other. The equivalent residues lie in similar positions in the (apo) structures of *Af*βG and *Ao*βG, except that in *Af*βG Phe512 takes the place of Tyr511. For the sugar molecule in subsite −1 of 4iig (Fig. 2 ▸), extensive hydrogen bonds tether the glucose molecule as follows: O1 to Tyr248 OH (3.1 Å) and to Glu509 OE2 (2.5 Å), O2 to Asp280 OD2 (3.0 Å) and Arg156 NH1 (2.7 Å), O3 to Arg156 NH2 (2.7 Å), Lys189 NZ (2.9 Å) and His190 NE2 (2.9 Å), and O4 and O6 to Asp92 OD1 and OD2, respectively (both 2.6 Å). These residues are conserved in all three enzymes and occupy very similar orientations in the *Af*βG and *Ao*βG structures. The orientation of the −1 subsite sugar of thiocellobiose in PDB entry 4iih is shifted relative to the position of the −1 subsite glucose molecule in 4iig; it lies towards the +1 subsite of the latter and the +1 subsite sugar is oriented further away from Tyr248 OH. The +1 subsite sugars in each complex form only one hydrogen bond to a protein side chain [glucose O6 to Tyr248 OH (2.3 Å) in PDB entry 4iig, thiocellobiose O3′ to Arg98 NH1 (2.9 Å) in PDB entry 4iih], and both of these side chains occupy a similar position in the structures of *Af*βG and *Ao*βG. Substrate-specificity studies with AoβG have demonstrated a tight specificity for β-d-glucopyranoside in the −1 subsite, with a much broader tolerance at the +1 subsite with similar catalytic efficiencies for glucose-β-1,2-, β-1,3-, β-1,4- and β-1,6-linked glucose and *p*-nitrophenyl-β-d-glucopyranoside (Langston *et al.*, 2006 ▸).

### 3.4. Extensive N-glycans; similarities to *Aa*βG glycans   

**Figure 3:**
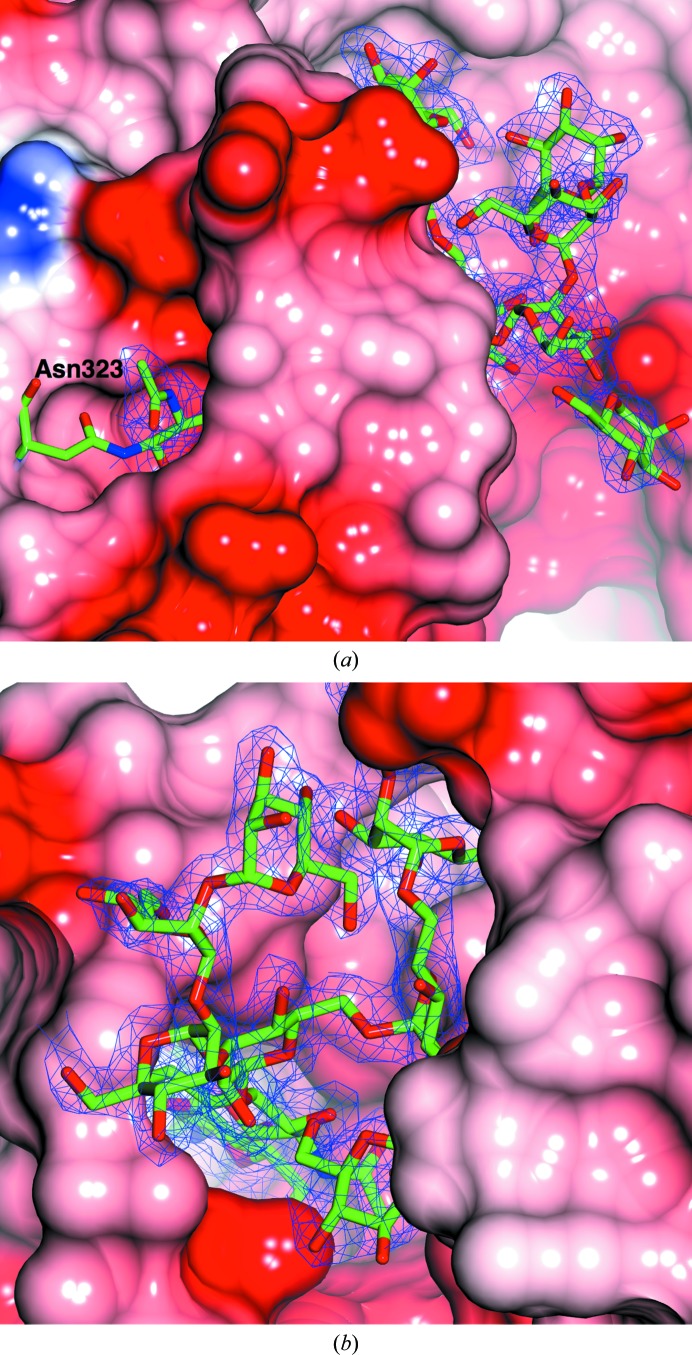
The electron density for the glycosylation tree attached to Asn323 in *Af*βG shown from two different perspectives. In (*a*) the first part of the tree is buried within a pocket of the protein. In (*b*) Asn323 is at the base of the pocket. There is well ordered density for all of the sugars. The maximum-likelihood map was contoured at the 1σ level.

**Figure 4:**
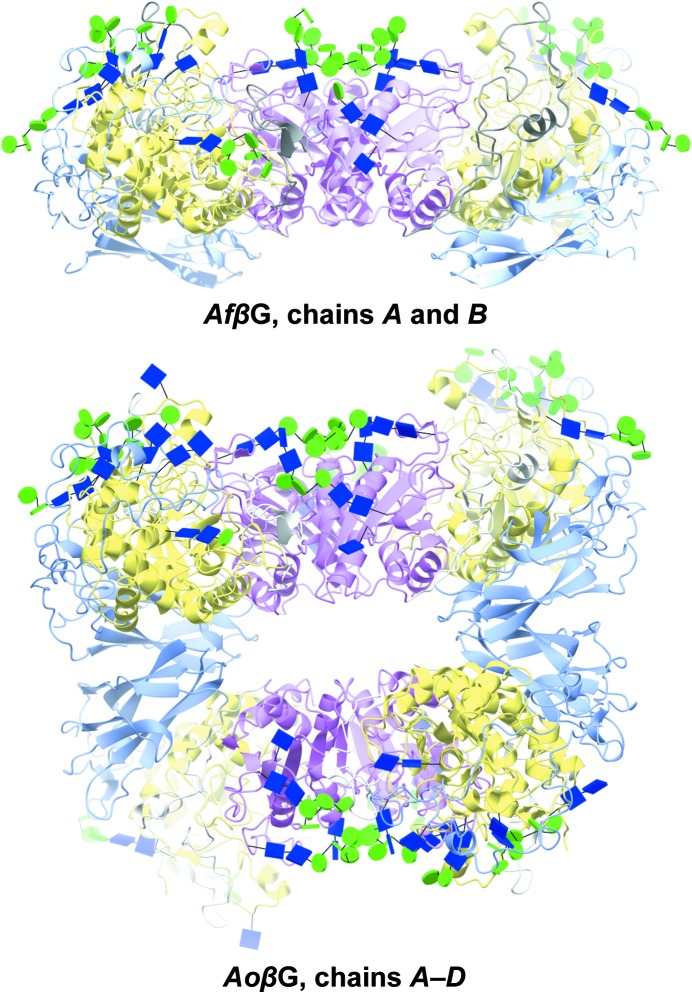
Three-dimensional fold, domain organization and asymmetric unit packing of *Af*βG and *Ao*βG. Both enzymes have three domains (A, yellow; B, pink; C, light blue), with a dimer being the preferred biological arrangement. *Ao*βG has two dimers in the asymmetric unit, with all of the sugars facing opposite sides. The sugars are shown as glycoblocks, with blue squares for *N*-acetyl-β-d-glucosamine and green circles for d-mannopyranose.

**Figure 5:**
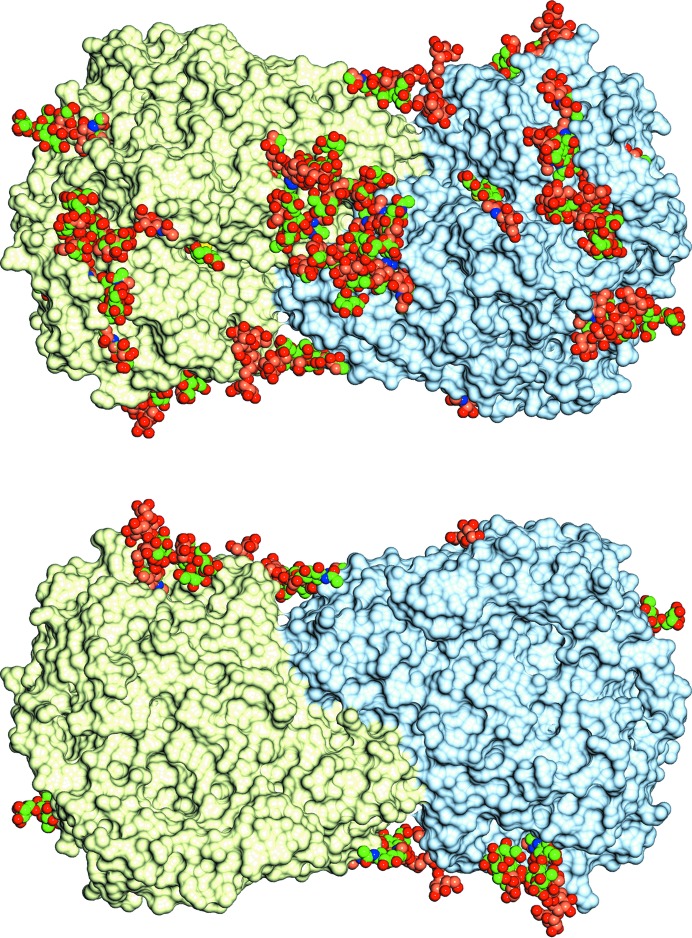
The glycosylation sites in *Af*βG and *Aa*βG with the sugars shown in space-filling representation, with the C atoms coloured brown for *Aa*βG and green for *Af*βG. The surface is that of *Af*βG coloured by chain. The two views are from opposite sides of the dimer and emphasize how the glycosylation trees are all located on just one side.

**Figure 6:**
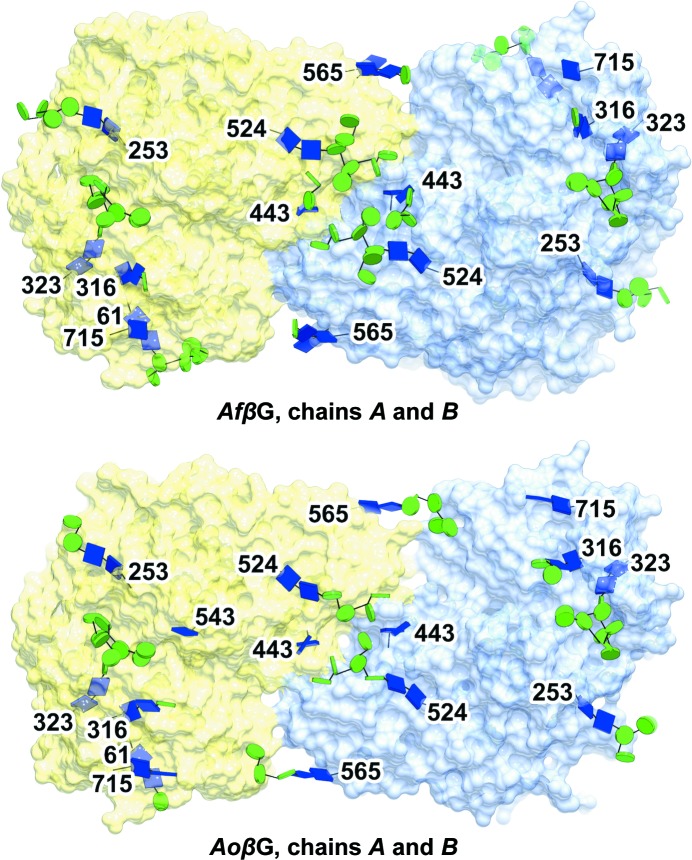
N-Glycosylation across *Af*βG and *Ao*βG. In both enzymes the abundant N-glycans all lie on one side of the molecular surface. Blue square, *N*-acetyl-β-d-glucosamine. Green circle, d-mannopyranose. Chain *A*, yellow. Chain *B*, light blue. The sugars are shown in the same representation as in Fig. 4 ▸.

The quality of the electron density for the sugars is typified by the structure of the glycosylation tree on Asn323 in *Af*βG (Fig. 3 ▸). There are 13 potential *N*-glycosylation sites in the amino-acid sequences of each of *Af*βG and *Ao*βG, with nine and ten of them being occupied in their respective crystal structures; we have modelled 45 and 46 sugars in chains *A* and *B*, respectively, for *Af*βG, whilst *Ao*βG has 40, 42, 41 and 34 sugars modelled in chains *A*, *B*, *C* and *D*, respectively. In Fig. 4 ▸, the glycosylation sites are shown in a novel representation by saccharide type as recently implemented in *CCP*4*mg* (Mc­Nicholas *et al.*, 2011 ▸). The organization of the N-glycan trees in both structures resembles that reported for *Aa*βG (Suzuki *et al.*, 2013 ▸), with high-mannose trees of similar lengths branching from structurally conserved sites (Tables 2 ▸ and 3 ▸), with the exceptions that *Af*βG lacks a glycan at Asn212 and that *Af*βG and *Ao*βG have an additional GlcNAc bound to Asn543. The glycosylation sites are located in domains A and B only, and predominantly on the opposite side of the molecule with respect to the FnIII domain, with the exception of the tree that starts at Asn253 and extends past a loop of domain C near the C-terminus. The distribution of sugars across both the *Af*βG and the *Ao*βG molecules is uneven, and the crystal packing in both X-ray structures features a large solvent channel (Fig. 4 ▸) lacking oligosaccharide decorations. This is enclosed between the two dimers of the asymmetric unit in the *Ao*βG structure, whilst the sugars are on the outer surface. The asymmetric distribution of the sugars on the surface of the dimer is strikingly evident in Fig. 5 ▸, where the sugars are shown in conventional space-filling format for *Af*βG and the published *Aa*βG structure (PDB entry 4iih), and Fig. 6 ▸, where they are shown in the novel glycoblock style used in Fig. 4 ▸.

Intriguingly, those sites located on the protein surface (Asn543 and Asn715 in *Af*βG, Asn212 and Asn543 in *Ao*βG) appear to have been truncated to a single GlcNAc molecule linked to their corresponding Asn residue, despite no attempt having been made to remove external N-glycans enzymatically. In addition, the external site at Asn713 in *Ao*βG (equivalent to Asn715 in *Af*βG) remained uncleaved even though the first GlcNAc molecule is in a very similar position and orientation to its equivalents in the other two structures. Asn211, which is linked to a single GlcNAc in *Aa*βG, is not occupied in *Af*βG (equivalent residue Asn212); in contrast, the external Asn543 sites (in both *Af*βG and *Ao*βG) are found to be glycosylated but truncated, whereas the equivalent residue Asn542 in *Aa*βG is not glycosylated. A very similar result was reported for all of the deposited structures of *Aa*βG (Suzuki *et al.*, 2013 ▸), even though that enzyme had been deglycosylated as part of the sample-preparation protocol. This suggests that the external glycans may be labile to secreted glycosidases.

**Figure 7:**
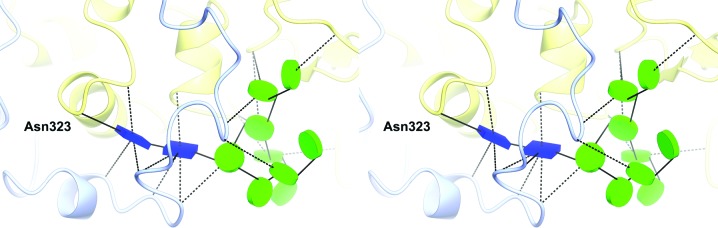
Schematic stereoview of the longest glycan in *Af*βG and its interactions. The glycan N-linked to Asn323 is a complete high-mannose tree (11 sugars) that establishes numerous hydrogen bonds to adjacent residues across domain A (yellow) and domain C (light blue). This glycan is very similar in *Ao*βG and *Aa*βG.

The largest N-glycan is linked to Asn323 in domain A, where it is visible as a complete nine-mannose tree in *Af*βG and *Ao*βG (chains *A* and *B* in the latter, chains *C* and *D* having seven and six mannoses, respectively). The sugars may play a stabilizing role through many hydrogen-bonding interactions with adjacent protein side chains of the N-terminal loop, the insertion loop and domain A (Fig. 7 ▸). A similar-length glycan (eight Man and two GlcNAc), which is also the largest visible glycan, was found linked to Asn322 in *Aa*βG. The N-glycans at Asn253 in *Af*βG and *Ao*βG and at Asn252 in *Aa*βG are generally visible beyond the first mannose and, apart from that in *Aa*βG, are able to establish hydrogen bonds to the protein downstream of the 1,3 branch, with a loop extending from the FnIII domain and, in *Af*βG, also with the insertion loop close to where it rejoins the FnIII domain. Similarly, the N-glycans at Asn565 in *Ao*βG and Asn564 in *Aa*βG exhibit hydrogen-bonding interactions with amino-acid residues in the adjacent chain, whereas in *Af*βG there is no density for the glycan downstream of the first mannose. The glycosylation trees at residues 524 (in *Ao*βG) and 523 (in *Aa*βG) exhibit hydrogen bonding to amino-acid residues across the subunit interface within the dimer interface between chains *A* and *B*. In *Ao*βG these glycans also interact with sugar atoms (on glycans bound to Asn524 and 443 of the dimer pair). In *Af*βG the equivalent sugars are not close enough to bind directly, but there are hydrogen bonds to bridging waters between the glycans on Asn524 on one subunit and Asn443 on the other. There is a further glycan tree of moderate length at Asn61 in *Af*βG (seven) and *Aa*βG (six), which forms hydrogen bonds to the loop between domains A and B.

**Figure 8:**
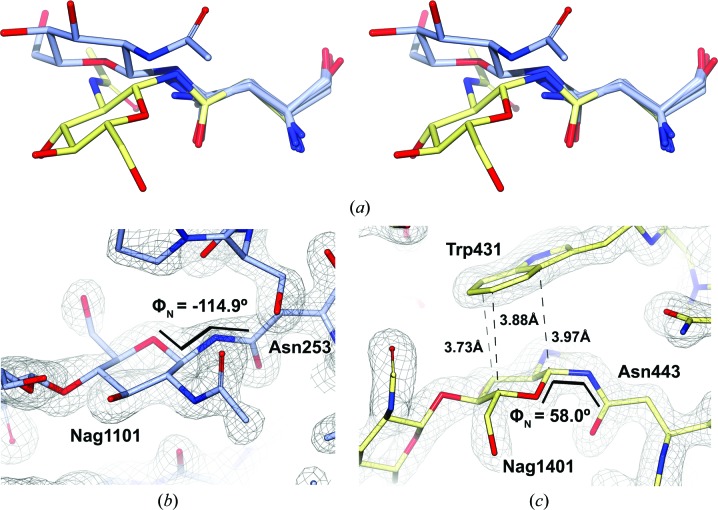
GlcNAc–Asn linkages in the two energy minima as found in the *Af*βG structure. (*a*) Stereoview of all superposed GlcNAc–Asn sites for chain *A*. For clarity reasons, Asn residues in other rotamer forms are omitted, and only one representative GlcNAc is shown for the two conformations (1101, with blue C atoms, and 1401, with yellow C atoms). (*b*) Nag1101–Asn253 as a representative of the most frequent, lowest-energy linkage conformation. (*c*) Nag1401–Asn443 in the secondary energy minimum described by Imberty & Perez (1995 ▸), with Trp431 taking part in CH–π stacking interactions with the apolar face of Nag1401. The electron-density maps shown here were calculated from 2*mF*_o_ − *DF*_c_ coefficients and contoured at 2σ. As they both show similar values (∼180°), ψ_N_ torsions are not depicted in (*b*) and (*c*).

While all other sites show GlcNAc–Asn glycopeptide linkages in the most commonly found conformation, *i.e.* with linkage torsions −140° < φ_N_ < −60° and ψ_N_ ≃ 180° (coincident with the absolute energy minimum; see Fig. 8 ▸ *b* for an example), the Nag1401–Asn443 linkage appears in a second, much less probable energy minimum (Imberty & Perez, 1995 ▸) which is stabilized by a CH–π interaction between the benzene ring of Trp431 and the apolar side of Nag1401 (Fig. 8 ▸ *c*). While the φ_N_ torsion is remarkably different for both minima, ψ_N_ remains at values of around 180°. Interestingly, this flipped Asn–GlcNAc linkage conformation is fully conserved in *Af*βG, *Ao*βG (CH–π interaction also with a Trp residue) and *Aa*βG, where a Tyr residue occupies the space near the apolar face of the carbohydrate. This amino-acid substitution preserves the character of the residue, allowing the same interaction to take place. It has recently been reported that Trp and Tyr together account for more than 80% of reported protein–carbohydrate CH–π stacking interactions (Hudson *et al.*, 2015 ▸).

All of the sugars composing the trees are in the expected low-energy ^4^ *C*_1_ conformation, with a mean puckering amplitude (Cremer & Pople, 1975 ▸) of 0.56 Å. This is in marked contrast to a significant proportion of the glycosylated structures in the PDB (Agirre, Davies *et al.*, 2015 ▸), where the use of conventional protocols in modelling and refinement has led to many of the pyranose rings being in higher energy conformations. For example, about 10% of the sugars were modelled in high-energy conformations in the coordinate sets deposited for *Aa*βG (Suzuki *et al.*, 2013 ▸).

## 4. Conclusion   

### 4.1. The importance of β-d-glucosidases in industrial biotechnology   

β-d-Glucosidases have been described as a ‘bottleneck’ in the efficient conversion of lignocellulosic biomass to simple sugars (Sørensen *et al.*, 2013 ▸; Singhania *et al.*, 2013 ▸). They act to relieve cellobiose inhibition of cellobiohydrolases and endoglucanases. However, β-d-glucosidases themselves are product-inhibited. The *A. oryzae* and *A. fumigatus* enzymes discussed here have an apparent *K*_i_ for glucose of 3.3 and 1.1 m*M*, respectively, which is typical for fungal β-d-glucosid­ases from GH3 (Bohlin *et al.*, 2010 ▸). Considering that glucose concentrations during industrially relevant biomass hydrolysis conditions rapidly reach 200 m*M* or greater, it is obvious that β-d-glucosidases such as these will be operating under conditions of substantial product inhibition during much of the hydrolysis time course. This necessitates the addition of higher levels of β-d-glucosidase to hydrolyse cellobiose than would be necessary in the absence of product inhibition (Bohlin *et al.*, 2013 ▸). A simple solution is simultaneous saccharification and fermentation wherein glucose is rapidly removed; however, the low temperatures at which current commercial fermentative organisms operate do not take full advantage of the higher temperature optimum of the hydrolytic enzymes and the trade-off is typically not in favour of this strategy, particularly with more recent commercial enzyme preparations (Ask *et al.*, 2012 ▸; Wirawan *et al.*, 2012 ▸; Cannella & Jørgensen, 2014 ▸; Agrawal *et al.*, 2015 ▸). Another possible solution is the use of certain GH1 family β-d-glucosidases with a much higher apparent *K*_i_ for glucose; however, many if not most of these have poor activity on cellobiose, although exceptions have been reported (Pei *et al.*, 2012 ▸; Cota *et al.*, 2015 ▸). A partial solution is to use enzymes with high catalytic efficiency such as *Af*βG, which at 50°C has a *k*_cat_ more than twice that of *Ao*βG and 37% higher than that of the *A. niger* β-d-glucosidase found in the commonly used β-d-glucosidase source Novozyme 188 (Bohlin *et al.*, 2010 ▸). Furthermore, the *A. fumigatus* enzyme retains most of its activity at 65°C for at least 19 h, whereas both the *A. oryzae* and *A. niger* enzymes retain activity for less than 2 h at this temperature (Kim *et al.*, 2007 ▸). Consequently, an even higher *k*_cat_ advantage can be achieved for *Af*βG at the elevated temperatures that are considered to be desirable for industrial biomass hydrolysis. The commercial importance of *Af*βG is underscored by the approximately 100 different patents and patent applications that reference it, dating back to 2005 (Harris & Golightly, 2005 ▸) and spanning nearly a decade.

The nature of the thermal stability differences is likely to be complex and multifactorial. One factor that directly affects irreversible protein denaturation is asparagine deamidation. Asparagine residues followed by glycines have been shown to be particularly susceptible to deamidation, and those followed by histidine and serine also exhibit significant deamidation within a short time (Robinson, 2002 ▸). However, given that the two proteins have a similar number of asparagine residues (50 in *Af*βG and 52 in *Ao*βG), we can rule out deamidation as a cause of the difference in stability.

Ion pairs have been implicated in thermal stability in many studies (Kumar & Nussinov, 1999 ▸; Barlow & Thornton, 1983 ▸). Analysis of the ion-pair patterns in the two enzymes reveals a few differences owing to variations in amino-acid sequence, which will affect both the intramolecular and intermolecular stability of the enzymes. Within the protomer, *Af*βG has two extra ion pairs, Lys151–Glu629 and Lys523–Asp516 (in addition, the former is relatively short at 2.6–2.9 Å), and the distance between residues 536 and 559 is shorter than that in *Ao*βG (2.9 Å compared with 3.0–3.7 Å). There are also closer ion-pair partner interactions in *Af*βG between Glu363 and residue 378 and between Arg387 and Glu131. *Af*βG is able to form two extra intermolecular salt bridges between Lys418 and Asp102 in both subunits of the dimer. *Ao*βG has an extra intradimer ion pair, Arg475–Asp384, but it is only close enough to qualify as a salt bridge in two of the four protomers (Arg475 in chains *B* and *D* to Asp384 in chains *A* and *C*, respectively).

Additional glycan-to-protein hydrogen bonding may enhance crystal packing in *Af*βG and *Ao*βG. The glycan decorations are located exclusively on domains A and B, and in the X-ray structures, the glycan at Asn253 extends towards and forms hydrogen bonds with the C domain. However, such interactions may not occur in solution if the FnIII domains occupy similar positions to that of their equivalent in the SAXS structure of *An*βgl1. Further studies with site-directed mutants would be required to establish the relative contributions of specific residues to the variation in the stability of these enzymes; these are beyond the bounds of this study.

### 4.2. Refinement protocols for sugars   

Ever since the inception of the CCP4 monomer library, harmonic torsion-angle restraints have been specified by a set of four ordered atoms plus an angle that can be either positive or negative, a standard deviation (tolerance) and a periodicity index, which accounts for the number of oscillations the function will make within a full rotation: for example, an initial value of 60° with periodicity 3 would allow values of around 60, −60 and 120° torsion. In the present situation, most torsion restraints for pyranose sugars contain a tolerance of 20°, which is a rather high value considering that smaller combined changes in the periods for the ring torsions could already force a different conformation. On top of this, the harmonic nature of the restraints – those with a periodicity index higher than 1 – makes them suitable for tolerating rather than enforcing multiple conformations that are by no means equiprobable. Most cyclic compounds have a clear preference, with high-energy barriers separating this conformation from the rest.

Higher-energy conformations in the most abundant pyran­ose sugars are very infrequent, thus a structure of a distorted sugar must only be modelled when it is supported by a clear chemical environment and complete unambiguous density. Taking into account that the theoretical purpose of torsion-angle restraints is to enforce a certain conformation upon a model that is being refined against poor or incomplete data, keeping them as they are now for pyranoses is inappropriate. Therefore, we propose that higher-energy conformations be treated as exceptions akin to Ramachandran outliers in protein model building – and even reported in Table 1 – and that torsion restraints should enforce the lowest energy, highest probability conformation of the pyranose ring where required. This can be accomplished either by reducing their periodicity to 1 or modifying existing refinement software to ignore their periodicity.

Beyond the structure determination and analysis of key enzymes in modern biotechnology, a major component of this study is the development and imposition of appropriate protocols for the modelling and refinement of pyranose sugars, especially those involved in glycosylation trees. All of the pyranoses in the two enzymes (91 for *Af*βG and 157 for *Ao*βG) were initially modelled and refined with suitable restraints to maintain them all in the preferred minimum-energy ^4^ *C*_1_ chair conformation (there are no l-pyranosides in these structures which would be expected to instead have the ^1^ *C*_4_ conformation). Sugars refined in their minimum-energy conformer are in marked contrast to those in many of the glycoprotein structures deposited in the PDB (Agirre, Davies *et al.*, 2015 ▸) where the lack of suitable restraints or in some cases the imposition of inappropriate restraints has resulted in many such sugars being in higher-energy conformations without good experimental data to support such anomalies. We propose that the protocols described here should be applied to the future refinement of at least N-glycosylation trees, and probably most other sugars, and could indeed also be used to remediate the present contents of the PDB where X-ray data have been deposited.

## Supplementary Material

PDB reference: *A. fumigatus* GH3, 5fji

PDB reference: *A. oryzae* GH3, 5fjj

## Figures and Tables

**Table 1:** X-ray data and refinement statistics Values in parentheses are for the high-resolution outer shell.

	*Af*βG	*Ao*βG
Space group	*P*2_1_2_1_2_1_	*P*2_1_2_1_2_1_
Unit-cell parameters (Å)	*a* = 88.5, *b* = 129.7, *c* = 217.7	*a* = 139.0, *b* = 141.5, *c* = 193.3
Data processing		
Resolution range (Å)	111–1.95 (2.0–1.95)	114–1.95 (2.0–1.95)
*R*_merge_	0.076 (0.44)	0.140 (0.84)
*R*_p.i.m._	0.070 (0.421)	0.076 (0.584)
CC_1/2_	0.991 (0.687)	0.995 (0.773)
〈*I*〉/〈σ(*I*)〉	8.3 (2.2)	17.1 (1.9)
Completeness (%)	96.8 (91.9)	97.8 (94.8)
Multiplicity	2.9 (2.6)	6.7 (4.9)
Model refinement		
No. of reflections used	167784	256551
No. of reflections in *R*_free_ set	11645	13558
*R*_cryst_/*R*_free_	0.15/0.17	0.22/0.25
No. of protein protomers	2	4
No. of protein atoms	13089	25943
No. of sugar atoms	1122	1948
No. of sugar monomers	91	157
No. of ligand atoms	191 (156 EDO†, 35 IMD‡)	48 (35 PEG§, 4 Cl^−^, 4 Mg^2+^, 5 PO_4_^3−^)
No. of water molecules	1527	2117
R.m.s.d., bonds (Å)	0.019	0.014
R.m.s.d., angles (°)	1.880	1.632
Mean *B* values (Å^2^)		
Protein	21	30
Sugar	38	42
Ligand	42	43
Water	32	33
Ramachandran plot¶ (%)		
Favoured	97.7	96.9
Allowed	2.3	3.1
Disallowed	0.0	0.0
Pyranose conformations (total/percentage)		
Lowest energy conformation	91/100	157/100
Higher energy conformations	0.0/0	0.0/0
PDB code	5fji	5fjj

† Ethylene glycol.

‡ Imidazole.

§ Polyethylene glycol.

¶ Calculated using *RAMPAGE* in *CCP*4.

**Table 2:** *Af*βG glycan descriptions

Chain *A*	Man-α1,2–Man-α1,3–Man-α1,6–(Man-α1,3–)Man-β1,4–GlcNAc-β1,4–GlcNAc-β–Asn61
	Man-α1,2–Man-α1,2–Man-α1,3–Man-β1,4–GlcNAc-β1,4–GlcNAc-β–Asn253
	Man-β1,4–GlcNAc-β1,4–GlcNAc-β–Asn316
	Man-α1,2–Man-α1,6–(Man-α1,2–Man-α1,3–)Man-α1,6–(Man-α1,2–Man-α1,2–Man-α1,3–)Man-β1,4–GlcNAc-β1,4–GlcNAc-β–Asn323
	Man-α1,3–Man-β1,4–GlcNAc-β1,4–GlcNAc-β–Asn443
	Man-α1,2–Man-α1,6–(Man-α1,3–)Man-α1,6–(Man-α1,2–Man-α1,3–)Man-β1,4–GlcNAc-β1,4–GlcNAc-β–Asn524
	GlcNAc-β–Asn543
	Man-β1,4–GlcNAc-β1,4–GlcNAc-β–Asn565
	GlcNAc-β–Asn715
Chain *B*	Man-α1,2–Man-α1,3–Man-α1,6–(Man-α1,3–)Man-β1,4–GlcNAc-β1,4–GlcNAc-β–Asn61
	Man-α1,2–Man-α1,2–Man-α1,3–Man-β1,4–GlcNAc-β1,4–GlcNAc-β–Asn253
	Man-β1,4–GlcNAc-β1,4–GlcNAc-β–Asn316
	Man-α1,2–Man-α1,6–(Man-α1,2–Man-α1,3–)Man-α1,6–(Man-α1,2–Man-α1,2–Man-α1,3–)Man-β1,4–GlcNAc-β1,4–GlcNAc-β–Asn323
	Man-α1,2–Man-α1,3–Man-β1,4–GlcNAc-β1,4–GlcNAc-β–Asn443
	Man-α1,2–Man-α1,6–(Man-α1,3–)Man-α1,6–(Man-α1,2–Man-α1,3–)Man-β1,4–GlcNAc-β1,4–GlcNAc-β–Asn524
	GlcNAc-β–Asn543
	Man-β1,4–GlcNAc-β1,4–GlcNAc-β–Asn565
	GlcNAc-β–Asn715

**Table 3:** *Ao*βG glycan descriptions

Chain *A*	Man-β1,4–GlcNAc-β1,4–GlcNAc-β–Asn62
	GlcNAc-β–Asn212
	Man-α1,6–Man-β1,4–GlcNAc-β1,4–GlcNAc-β–Asn253
	Man-β1,4–GlcNAc-β1,4–GlcNAc-β–Asn316
	Man-α1,2–Man-α1,6–(Man-α1,2–Man-α1,3–)Man-α1,6–(Man-α1,2–Man-α1,2–Man-α1,3–)Man-β1,4–GlcNAc-β1,4–GlcNAc-β–Asn323
	GlcNAc-β1,4–GlcNAc-β–Asn443
	Man-α1,2–Man-α1,6–Man-α1,6–Man-β1,4–GlcNAc-β1,4–GlcNAc-β–Asn524
	GlcNAc-β–Asn543
	Man-α1,2–Man-α1,3–Man-α1,6–(Man-α1,3–)Man-β1,4–GlcNAc-β1,4–GlcNAc-β–Asn565
	GlcNAc-β1,4–GlcNAc-β–Asn713
Chain *B*	Man-α1,6–Man-β1,4–GlcNAc-β1,4–GlcNAc-β–Asn62
	Man-α1,6–(Man-α1,2–Man-α1,3–)Man-β1,4–GlcNAc-β1,4–GlcNAc-β–Asn253
	Man-α1,6–Man-β1,4–GlcNAc-β1,4–GlcNAc-β–Asn316
	Man-α1,2–Man-α1,6–(Man-α1,2–Man-α1,3–)Man-α1,6–(Man-α1,2–Man-α1,2–Man-α1,3–)Man-β1,4–GlcNAc-β1,4–GlcNAc-β–Asn323
	GlcNAc-β1,4–GlcNAc-β–Asn443
	Man-α1,2–Man-α1,6–(Man-α1,3–)Man-α1,6–Man-β1,4–GlcNAc-β1,4–GlcNAc-β–Asn524
	GlcNAc-β–Asn543
	Man-α1,3–Man-α1,6–Man-β1,4–GlcNAc-β1,4–GlcNAc-β–Asn565
	GlcNAc-β1,4–GlcNAc-β–Asn713
Chain *C*	Man-α1,6–Man-β1,4–GlcNAc-β1,4–GlcNAc-β–Asn62
	GlcNAc-β–Asn212
	Man-α1,2–Man-α1,3–Man-β1,4–GlcNAc-β1,4–GlcNAc-β–Asn253
	Man-β1,4–GlcNAc-β1,4–GlcNAc-β–Asn316
	Man-α1,2–Man-α1,6–(Man-α1,2–Man-α1,3–)Man-α1,6–(Man-α1,3–)Man-β1,4–GlcNAc-β1,4–GlcNAc-β–Asn323
	GlcNAc-β1,4–GlcNAc-β–Asn443
	Man-α1,2–Man-α1,6–(Man-α1,3–)Man-α1,6–Man-β1,4–GlcNAc-β1,4–GlcNAc-β–Asn524
	GlcNAc-β–Asn543
	Man-α1,2–Man-α1,6–(Man-α1,3–)Man-α1,6–Man-β1,4–GlcNAc-β1,4–GlcNAc-β–Asn565
	GlcNAc-β1,4–GlcNAc-β–Asn713
Chain *D*	GlcNAc-β1,4–GlcNAc-β–Asn62
	GlcNAc-β1,4–GlcNAc-β–Asn253
	Man-α1,3–Man-β1,4–GlcNAc-β1,4–GlcNAc-β–Asn316
	Man-α1,2–Man-α1,6–(Man-α1,2–Man-α1,3–)Man-α1,6–Man-β1,4–GlcNAc-β1,4–GlcNAc-β–Asn323
	GlcNAc-β1,4–GlcNAc-β–Asn443
	Man-α1,2–Man-α1,6–(Man-α1,3–)Man-α1,6–Man-β1,4–GlcNAc-β1,4–GlcNAc-β–Asn524
	GlcNAc-β–Asn543
	Man-α1,2–Man-α1,3–Man-α1,6–(Man-α1,3–)Man-β1,4–GlcNAc-β1,4–GlcNAc-β–Asn565
	GlcNAc-β–Asn713
